# The dependence of maximum oxygen uptake and utilization (*V̇*O_2_max) on hemoglobin‐oxygen affinity and altitude

**DOI:** 10.14814/phy2.15806

**Published:** 2023-08-31

**Authors:** Kevin L. Webb, Michael J. Joyner, Chad C. Wiggins, Timothy W. Secomb, Tuhin K. Roy

**Affiliations:** ^1^ Department of Anesthesiology and Perioperative Medicine Mayo Clinic Rochester Minnesota USA; ^2^ Department of Physiology and Biomedical Engineering Mayo Clinic Rochester Minnesota USA; ^3^ Department of Physiology University of Arizona Tucson Arizona USA

## Abstract

Oxygen transport from the lungs to peripheral tissue is dependent on the affinity of hemoglobin for oxygen. Recent experimental data have suggested that the maximum human capacity for oxygen uptake and utilization (*V̇*O_2_max) at sea level and altitude (~3000 m) is sensitive to alterations in hemoglobin‐oxygen affinity. However, the effect of such alterations on *V̇*O_2_max at extreme altitudes remains largely unknown due to the rarity of mutations affecting hemoglobin‐oxygen affinity. This work uses a mathematical model that couples pulmonary oxygen uptake with systemic oxygen utilization under conditions of high metabolic demand to investigate the effect of hemoglobin‐oxygen affinity on *V̇*O_2_max as a function of altitude. The model includes the effects of both diffusive and convective limitations on oxygen transport. Pulmonary oxygen uptake is calculated using a spatially‐distributed model that accounts for the effects of hematocrit and hemoglobin‐oxygen affinity. Systemic oxygen utilization is calculated assuming Michaelis–Menten kinetics. The pulmonary and systemic model components are solved iteratively to compute predicted arterial and venous oxygen levels. Values of *V̇*O_2_max are predicted for several values of hemoglobin‐oxygen affinity and hemoglobin concentration based on data from humans with hemoglobin mutations. The model predicts that increased hemoglobin‐oxygen affinity leads to increased *V̇*O_2_max at altitudes above ~4500 m.

## 1 INTRODUCTION

Human activity is drastically constrained at extreme altitudes due to low environmental oxygen availability. Few humans can reach the highest elevation on earth, the summit of Mt. Everest (~8850 m), without supplemental oxygen, and those who do are limited to such a degree that a slow uphill walk approaches the maximum capacity for oxygen uptake and utilization (*V̇*O_2_max).

Over millennia, several animal species have adapted to life at extreme altitudes, in part due to a high hemoglobin‐oxygen affinity (Storz, 2007; Storz et al., 2010). The most common metric of hemoglobin‐oxygen affinity is *P*_50_, the oxygen tension at which 50% of hemoglobin is saturated. A low *P*_50_ corresponds to a high hemoglobin‐oxygen binding affinity and vice‐versa. Recent investigation has highlighted a rare human population with hemoglobin mutations causing high hemoglobin‐oxygen affinity (low *P*_50_) (Charache et al., 1966; Thom et al., 2013). This human population has shown remarkable maintenance of exercise tolerance during normobaric hypoxia and at terrestrial high altitude (~3000 m) (Dominelli et al., 2020; Hebbel et al., 1978; Webb, Dominelli, et al., 2022a), perhaps in part due to the elevated hemoglobin values observed. Furthermore, there has been growing interest regarding the effects of pharmacologically altering the *P*_50_ in healthy individuals with typical hemoglobin (Stewart et al., 2021, 2020). Despite hemoglobin function being central to oxygen transport and utilization, much remains unknown regarding the effects of an altered *P*_50_.

Changes in *P*_50_ can markedly influence pulmonary oxygen loading and peripheral offloading. For instance, a decrease in *P*_50_ may enhance pulmonary oxygen loading at the expense of blunted peripheral offloading. At sea level, where arterial blood is well‐oxygenated, a low *P*_50_ likely hinders peripheral offloading leading to several compensatory adaptations (i.e., enhanced red blood cell production and increased oxygen carrying capacity), as observed in humans with hemoglobin mutations (Webb, Dominelli, et al., 2022a). Yet at high and extreme altitudes, a low *P*_50_ likely improves arterial blood oxygenation with subsequent preservation of *V̇*O_2_max. However, experimental data regarding the influence of an altered *P*_50_ are largely limited to examination at high altitude conditions (~3000 m or 15% O_2_ in the laboratory setting). The question remains: how would humans with a low *P*_50_ tolerate extreme altitudes (Bencowitz et al., 1982)?

Because hemoglobin mutations are exceedingly rare and research sojourns to extreme altitudes are challenging to achieve, we present a theoretical model of oxygen transport to investigate the dependence of *V̇*O_2_max on *P*_50_ and altitude. We hypothesized that relative to a normal *P*_50_, a low *P*_50_ would result in a greater *V̇*O_2_max at high altitude and that this relationship would be further potentiated at extreme altitudes.

## 2 METHODS

**FIGURE 1:**
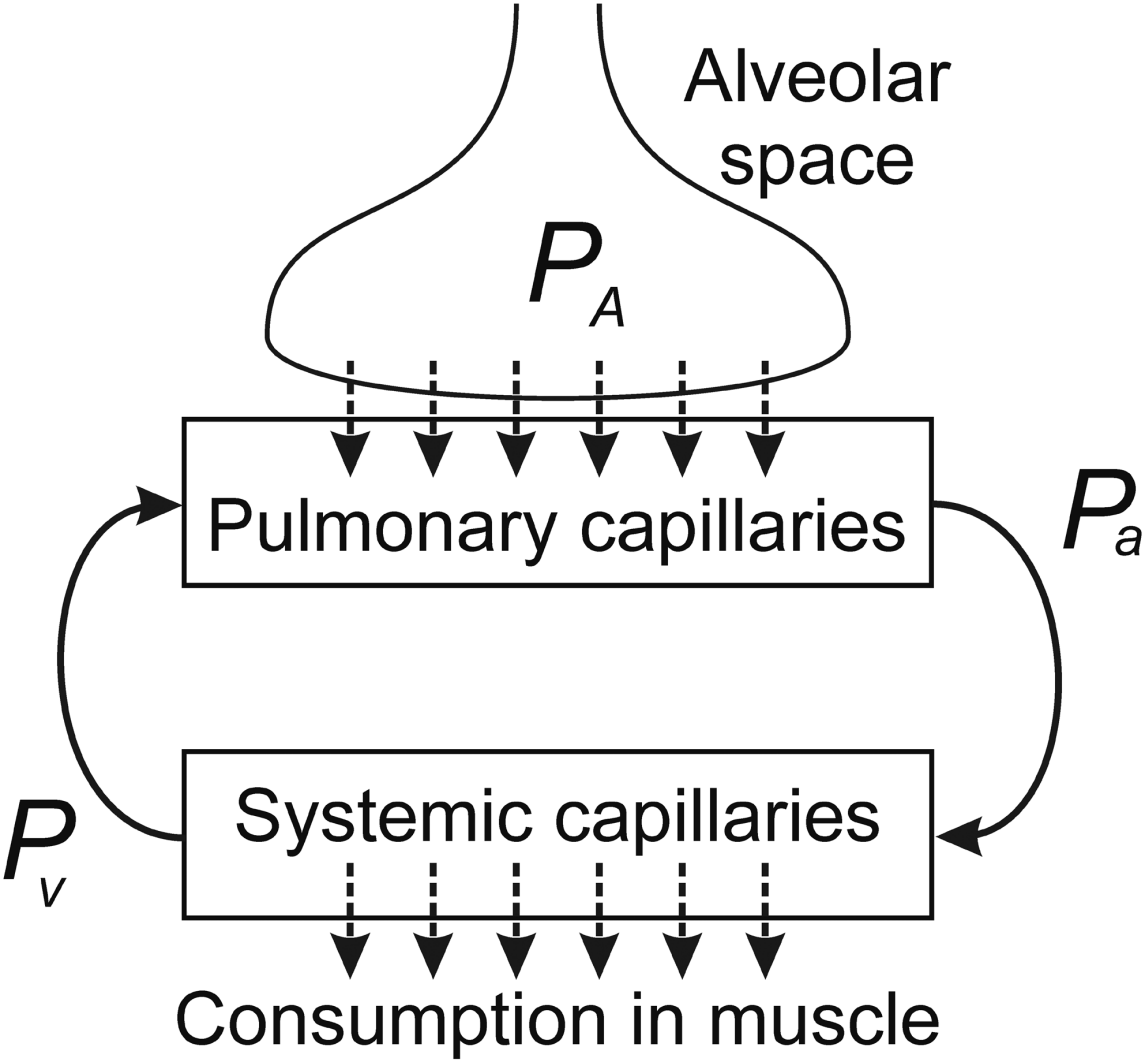
Schematic of modeling configuration, combining pulmonary oxygen uptake and systemic oxygen utilization. The dashed arrows indicate diffusive oxygen transport, and the solid arrows indicate convective oxygen transport within the blood. *P*_A_, alveolar oxygen tension; *P*_a_, arterial oxygen tension; *P*_v_, venous oxygen tension.

The *V̇*O_2_max achieved in any given situation reflects limitations in both pulmonary oxygen uptake and systemic oxygen utilization. We therefore simulated whole‐body oxygen uptake and utilization to investigate the variation in *V̇*O_2_max as a function of altitude in humans with normal and altered hemoglobin‐oxygen affinity. A mathematical model for oxygen uptake in the lung was combined with a model for oxygen utilization in the systemic circulation, as indicated in Figure 1, to predict arterial and venous oxygen tensions and oxygen consumption rate.

To represent conditions of maximal exercise, the tissue oxygen demand and cardiac output were selected in accordance with observed sea‐level *V̇*O_2_max values (see “Systemic oxygen utilization model” below). Arterial and venous oxygen tensions were then predicted at progressively lower atmospheric pressures (i.e., increasing altitude). The resulting arteriovenous oxygen content differences were used to estimate oxygen consumption as a function of altitude and as a measure of functional capacity. To investigate the effects of hemoglobin‐oxygen affinity on *V̇*O_2_max, calculations were performed for three cases of *P*_50_ (low, normal, and high) with a range of potential changes in hemoglobin concentration as a function of altitude (Table 1). Further details of the mathematical model are given below, and parameter values are provided in Table 2.

**TABLE 1:** Cases considered for modeling *V̇*O_2_max as a function of altitude. Data depict cases considered for variation in hemoglobin‐oxygen affinity (*P*_50_) and hemoglobin concentration in humans during sojourn to extreme altitudes.

Normal hemoglobin‐oxygen affinity			High hemoglobin‐oxygen affinity			Low hemoglobin‐oxygen affinity		
	*P*_50_ (mmHg)	Hemoglobin concentration (g/dL)		*P*_50_ (mmHg)	Hemoglobin concentration (g/dL)		*P*_50_ (mmHg)	Hemoglobin concentration (g/dL)
Case 1	26.3, Ref. (20)	14.8, Ref. (17)	Case 1	15.6, Ref. (5)	14.8	Case 1	37	14.8
Case 2	26.3	14.8 → 19.9, Ref. (17)	Case 2	15.6	18.7, Ref. (21)	Case 2	37	12.0
Case 3	26.3 → 24.8, Ref. (17)	14.8	Case 3	15.6	18.7 → 19.9*	Case 3	37	12 → 16.2*
Case 4	26.3 → 24.8	14.8 → 19.9	Case 4	15.6 → 14.7*	18.7	Case 4	37 → 34.9*	12.0
			Case 5	15.6 → 14.7*	18.7 → 19.9*	Case 5	37 → 34.9*	12 → 16.2*

*Note*: Data were taken from experimental results when available. Arrows indicate changes in values from sea‐level to an altitude of ~8400 m Ref (17).

* Indicates data for case of high or low hemoglobin‐oxygen affinity was assumed to change proportionally to alterations observed during extreme altitude sojourn among humans with normal hemoglobin‐oxygen affinity.

**TABLE 2:** Parameter values used for oxygen transport calculations.

Parameter	Value	Units	Citation
Water vapor pressure $P_{w}$	47	mmHg	–
Respiratory quotient R	0.8	–	–
Michaelis constant for oxygen consumption $P_{0}$	10.5	mmHg	(Golub & Pittman, 2012)
Lung diffusing capacity $D_{LO_{2}}$	74	cm^3^ O_2_ min^−1^ mmHg^−1^	(Roy & Secomb, 2019)
Capillary length $L_{\mathrm{tot}}$	0.5	mm	–
Hill coefficient n	2.7	‐	(Hsia, 1998)

### 2.1 Pulmonary oxygen uptake model

The model for pulmonary oxygen uptake yields estimates of arterial oxygen tension $\left(P_{a}\right)$ for given values of alveolar oxygen tension $\left(P_{A}\right)$ and venous oxygen tension $\left(P_{v}\right)$. Oxygen uptake is calculated using a single‐compartment model that accounts for the effects of capillary diameter and hematocrit based on simplified capillary and erythrocyte geometry (Roy & Secomb, 2019). The assumed value for the lung diffusing capacity $D_{LO_{2}}$ (Table 2) was selected to account for the heterogeneity of perfusion present in the pulmonary circulation (Roy & Secomb, 2014). The diffusion of oxygen from alveoli into the blood during pulmonary capillary perfusion is represented by:

(1) $$QC_{0}\frac{\mathrm{dS}\left(P_{b}\right)}{\mathrm{dx}}=\frac{D_{LO_{2}}}{L_{\mathrm{tot}}}\left(P_{A}-P_{b}\right)$$

where $P_{b}$ is blood oxygen tension, $C_{0}$ is oxygen content of fully saturated blood (calculated as 1.34 × [Hb]), Q is mean capillary flow rate, x is distance, $S\left(P_{b}\right)$ describes the oxyhemoglobin dissociation curve, and $L_{\mathrm{tot}}$ is the total length of pulmonary capillaries. Setting t=x/L, where L is mean capillary length and $Q_{\mathrm{tot}}$ is the total blood flow (i.e., cardiac output), yields:

(2) $$\frac{dP_{b}}{\mathrm{dt}}=\frac{D_{LO_{2}}\left(P_{A}-P_{b}\right)}{Q_{\mathrm{tot}}C_{0}S^{\prime }\left(P_{b}\right)}$$

This equation was integrated from t=0 to t=1 with the initial condition $P_{b}\left(0\right)=P_{v}$, to obtain $P_{a}=P_{b}\left(1\right)$.

The cardiac output $Q_{\mathrm{tot}}$ (L/min) was estimated based on a published correlation for measurements made over a range of ${\dot{V}}_{O_{2}}$ values (L/min) by averaging the results from two different experimental techniques (Calbet & Boushel, 2015):

(3) $$Q_{\mathrm{tot}}=4.37+5.33{\dot{V}}_{O_{2}}$$

(4) $$Q_{\mathrm{tot}}=4.43+5.22{\dot{V}}_{O_{2}}$$

To facilitate the calculation of $P_{A}$ via the alveolar gas equation, data for subjects at altitude were obtained from measurements performed on climbers summiting Mt. Everest, with an altitude of ~8850 m (Grocott et al., 2009). Values of hemoglobin concentration, $P_{O_{2}}$, and $P_{CO_{2}}$ were obtained by digitizing Figure 2 of Grocott et al. (Grocott et al., 2009) and a linear regression was used to fit these data as a function of altitude. The value of $P_{50}$ was assumed to be 26.3 mmHg at sea level (see Table 1). To obtain an estimate of $P_{50}$ at the summit, values of $P_{O_{2}}$ and saturation as reported in table 2 of Grocott et al. (2009) were fit using a nonlinear regression and an assumed value of 2.7 for the Hill coefficient n:

(5) $$S\left(P\right)=\frac{P^{n}}{P^{n}+{P_{50}}^{n}}$$

**FIGURE 2:**
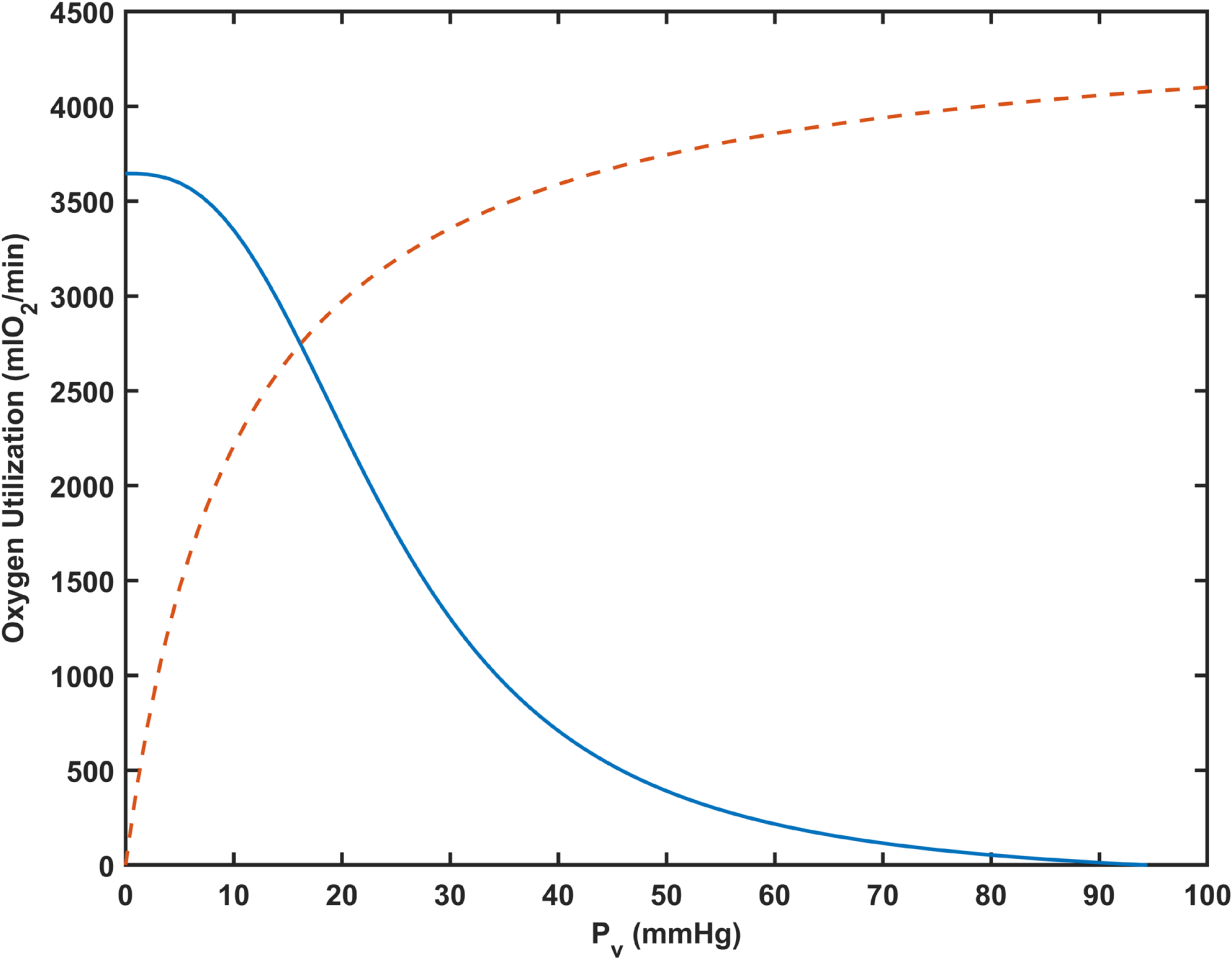
Example of predicted maximal oxygen utilization rate using the systemic model. Conditions correspond to atmospheric pressure at sea level with typical hemoglobin‐oxygen affinity and a hemoglobin concentration of 14.778 g/dL. Oxygen demand (M) was set as 4530 mL O_2_/min. The dashed line represents ${\dot{V}}_{O_{2}}$ calculated from Michaelis–Menten kinetics. The solid line represents ${\dot{V}}_{O_{2}}$ calculated from the Fick principle. Resulting predicted values at the intersection of the curves are ${\dot{V}}_{O_{2}}$ = 2750 mL O_2_/min and a venous partial pressure of oxygen $\left(P_{v}\right)$ of 16.2 mmHg. *P*_v_, venous partial pressure of oxygen.

Values of barometric pressure were calculated from West et al. (1999).

(6) $$P_{B}\left(a\right)=\exp\left(-0.00149a^{2}-0.1112a+6.63268\right)$$

where a is the altitude in kilometers. The simplified form of the alveolar gas equation was then used to calculate alveolar $P_{O_{2}}$ as a function of altitude:

(7) $$P_{IO_{2}}=F_{IO_{2}}\left(P_{B}\left(a\right)-P_{w}\right)$$

(8) $$P_{A}\left(a\right)=P_{IO_{2}}-\frac{P_{CO_{2}}\left(a\right)}{R}$$

where $P_{w}$ represents water vapor pressure and R represents the respiratory quotient (see Table 2). The values of $P_{CO_{2}}$ used in Equation 8, from Ref. (Grocott et al., 2009), were assumed to approximate the values under conditions of *V̇*O_2_max.

### 2.2 Systemic oxygen utilization model

The systemic model yields estimates of $P_{v}$ for given values of $P_{a}$ and tissue oxygen demand. According to the model, as altitude increases, levels of capillary $P_{O_{2}}$ are reduced, limiting the pressure gradient for diffusive transport to tissue, eventually resulting in tissue hypoxia. Under these conditions, oxygen consumption rate falls short of oxygen demand. The local rate of oxygen consumption is generally assumed to depend on tissue $P_{O_{2}}$ with Michaelis–Menten kinetics (Popel, 1989). Estimating distributions of tissue $P_{O_{2}}$ levels would require several additional assumptions regarding capillary density and oxygen transport properties. Thus, we employed a simplified approach, based on the assumption that $P_{v}$ can be used as an approximation for tissue $P_{O_{2}}$. In this approach, oxygen consumption is assumed to be a function of $P_{v}$ with Michaelis–Menten kinetics:

(9) $${\dot{V}}_{O_{2}}=M\frac{P_{v}}{P_{v}+P_{0}}$$

where M is oxygen demand is oxygen demand, which is calculated such that predicted ${\dot{V}}_{O_{2}}$ at sea level corresponded to a typical observed value of ${\dot{V}}_{O_{2}\max}$ = 2750 mL O_2_/min in healthy young adults (van der Steeg & Takken, 2021). The oxygen demand M represents mitochondrial oxygen consumption capacity under conditions of unlimited oxygen supply.

The model for systemic oxygen utilization uses a simplified approach, based on the assumption that $P_{v}$ can be used as an approximation for tissue $P_{O_{2}}$. In reality, steep gradients in in tissue oxygen tensions around capillaries are present at *V̇*O_2_max, such that tissue $P_{O_{2}}$ is less than local capillary $P_{O_{2}}$. Also, intravascular $P_{O_{2}}$ declines in the axial direction along capillaries, such that venous $P_{O_{2}}$ represents a lower bound on capillary $P_{O_{2}}$. From these considerations, it follows that $P_{v}$ represents an intermediate value within the range of tissue $P_{O_{2}}$ levels and can be used as an approximate estimate of tissue $P_{O_{2}}$. The advantage of this approach, termed Fick–Michaelis–Menten (FickMM), is that it provides an estimate that is independent of capillary density and geometric arrangement, which are highly variable and for which data are not generally available for human subjects.

According to the Fick principle, oxygen consumption rate must also satisfy:

(10) $${\dot{V}}_{O_{2}}=Q_{\mathrm{tot}}C_{0}\left(S\left(P_{a}\right)-S\left(P_{v}\right)\right)$$

where $S\left(P\right)$ describes the oxyhemoglobin dissociation curve and other quantities are defined in the main text. For any given set of conditions, the predicted values of ${\dot{V}}_{O_{2}}$ and $P_{v}$ correspond to the simultaneous solution of Equations 9 and 10, as shown graphically in Figure 2.

To evaluate the validity of approximating tissue $P_{O_{2}}$ by venous $P_{O_{2}}$, comparisons with a model using a conventional Krogh geometry and Michaelis–Menten kinetics were performed using the oxygen transport and geometric parameters described in Table 3. The calculations were performed by solving the radial diffusion equation in successive slices of a cylinder surrounding a central capillary, as described elsewhere (McGuire & Secomb, 2001). These simulations take into account the variations in tissue *P*O_2_ with axial and radial position in the Krogh cylinder, when calculating the overall rate of oxygen consumption. From the results, the venous oxygen tension exiting the muscle compartment was estimated for a range of *P*_a_, and for several capillary densities (McGuire & Secomb, 2001). The entire cardiac output was assumed to be directed to the muscle compartment.

**TABLE 3:** Assumed parameters and values used in the mathematical model.

Description	Parameter	Value	Units	Source
Sherwood number	Sh	2.5		(Hellums et al., 1996)
Plasma oxygen diffusivity	D_pl_	2.18E‐5	cm^2^ s^−1^	(Hellums et al., 1996)
Plasma oxygen solubility	α_pl_	2.82E‐5	mL O_2_ cm^−3^ mmHg^−1^	(Christoforides et al., 1969)
Tissue capillary radius	r_c_	2.5	μm	(Roy & Secomb, 2014)
Tissue oxygen diffusivity	D_t_	2.41E‐5	cm^2^ s^−1^	(Bentley et al., 1993)
Tissue oxygen solubility	α_t_	3.89E‐5	mL O_2_ cm^−3^ mmHg^−1^	(Bentley et al., 1993)

**FIGURE 3:**
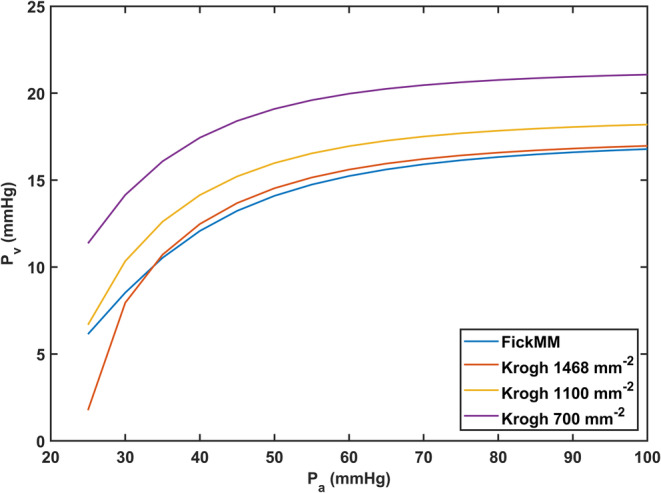
Estimates of venous oxygen tension (Pv) obtained using the Fick‐Michaelis Menten (FickMM) model as compared to using a Krogh model with Michaelis–Menten kinetics for muscle oxygen utilization. Results are presented as a function of arterial oxygen tension (*P*_a_) for various capillary densities (1468, 1100, and 700 mm^−2^) depicted in the figure legend. Computations were performed using the oxygen transport parameters in Table 3.

The results of these simulations (Figure 3) showed that the results for venous *P*O_2_ were similar to FickMM for capillary densities in the range of 1100–1468 mm^−2^. While lower values of capillary density have been reported (Klausen et al., 1981; Qu et al., 1997; Richardson, 1995), prior calculations by McGuire and Secomb (2003) demonstrate that higher values of capillary density (1100–1468 mm^−2^) are consistent with measured oxygen uptake and utilization rates, suggesting that histologically measured capillary densities may underestimate functional values in vivo. For lower capillary densities, the FickMM model would exaggerate the *V̇*O_2_ levels that could be achieved. Similar results were seen for simulations performed with high and low affinity hemoglobin variants (*P*_50_ = 15.6 and 37 mmHg).

Corresponding calculations with a lower capillary density and not all cardiac output going to the muscle would require a higher oxygen demand to match the values of *V̇*O_2_max assumed at sea level. If the Krogh model were used with a lower capillary density, then the predicted values of *V̇*O_2_max would be lower than those obtained with the FickMM model, but would show similar trends with altitude. The value of oxygen demand assumed for FickMM was calculated on the basis of the entire cardiac output being directed to the muscle compartment. Including effects of flow distribution to other organs would result in lower predicted *V̇*O_2_max values. For these reasons, the estimates of *V̇*O_2_max reported in the paper can be considered upper bounds.

The determinants of *V̇*O_2_max may be represented by plotting convective and diffusive limitations of O_2_ delivery as a function of venous *P*O_2_, in a graph referred to as a “Wagner diagram” (Poole et al., 2012; Wagner, 1996). The diffusive limitation on oxygen transport was estimated using the Krogh cylinder model, using assumed values of capillary density, capillary diameter, intracapillary diffusion resistance, and blood and plasma oxygen diffusivity and solubility:

(11) $$P_{v}={\dot{V}}_{O_{2}}\left[\frac{{r_{t}}^{2}-{r_{c}}^{2}}{K_{\mathrm{pl}}∙\mathrm{Sh}}-\frac{{r_{t}}^{2}-{r_{c}}^{2}-2{r_{t}}^{2}\ln\left(r_{t}/r_{c}\right)}{4K}\right]$$

where *r*_*c*_ is the capillary radius, *K* and *K*_pl_ are Krogh diffusion coefficients in the tissue and the plasma, and Sh is the Sherwood number representing intravascular diffusion resistance. The tissue cylinder radius *r*_*t*_ is computed based on an estimated capillary density obtained from Figure 3, such that the tissue *P*O_2_ matches the value obtained by the FickMM model. Convective oxygen delivery was calculated by the Fick principle as in Equation 10. Calculations were performed assuming that hemoglobin values did not change with altitude (Case 2) and that cardiac output was the same in all cases.

### 2.3 Cases considered

Cases of low *P*_50_, normal *P*_50_, and high *P*_50_ were investigated. The variation of hemoglobin parameters with altitude is not well established, particularly among humans with hemoglobin mutations. To encompass the likely range of variations in hemoglobin concentration and *P*_50_ with altitude, various cases indicated in Table 1 were considered for each hemoglobin variant. These cases include ones in which the $P_{50}$ was assumed to remain constant as a function of altitude, and others in which the $P_{50}$ was assumed to decrease with altitude, as was observed Grocott et al. (2009). Corresponding values of hemoglobin concentration were assumed either to be constant or to vary with altitude according to the ratio observed experimentally, subject to the maximum value (Grocott et al., 2009).

## 3 RESULTS

Model predictions of blood oxygenation and *V̇*O_2_max as a function of altitude are presented for cases of low, normal, and high *P*_50_, and for several different assumptions about the variations of *P*_50_ and hemoglobin concentration with altitude, as indicated in Table 1.

**FIGURE 4:**
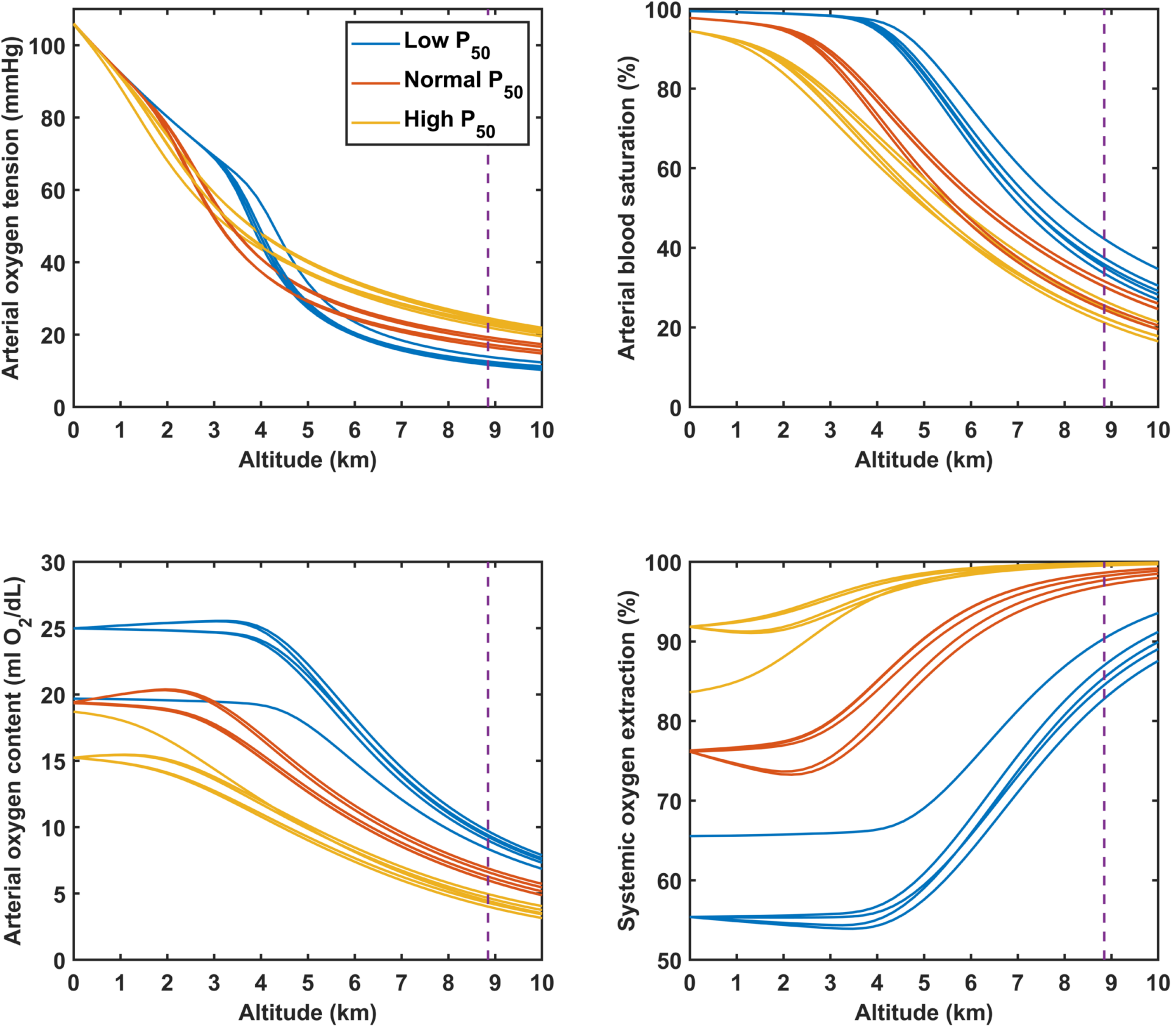
Predicted oxygen transport parameters as a function altitude for cases of low, normal, and high hemoglobin‐oxygen affinity. Data are depicted for several cases of hemoglobin‐oxygen affinity (low *P*_50_ in blue, normal *P*_50_ in red, and high *P*_50_ in yellow) with variable hemoglobin concentrations to account for potential differences in the hematological response to extreme altitude sojourn indicated in Table 1. These parameters are derived for a given tissue oxygen demand that corresponds with sea‐level maximum oxygen uptake and utilization (*V̇*O_2_max). The elevation associated with the summit of Everest is depicted by the dashed vertical line. Parameters corresponding to altitudes above 8400 m are derived from the extrapolation of oxygen transport parameters in Grocott et al. (2009). P_50_, oxygen tension at which 50% of hemoglobin is saturated with oxygen.

Profound blood gas alterations occur during human sojourn to extreme altitudes. Figure 4 displays predicted oxygen transport parameters for the three cases of *P*_50_ (low, normal, and high) as a function of altitude. The variation of arterial oxygen tension with increasing altitude is similar for all cases considered. However, arterial oxygen saturation and oxygen content are substantially increased at high and extreme altitudes for cases of low *P*_50_ compared to predicted values for cases of normal *P*_50_ and high *P*_50_.

**FIGURE 5:**
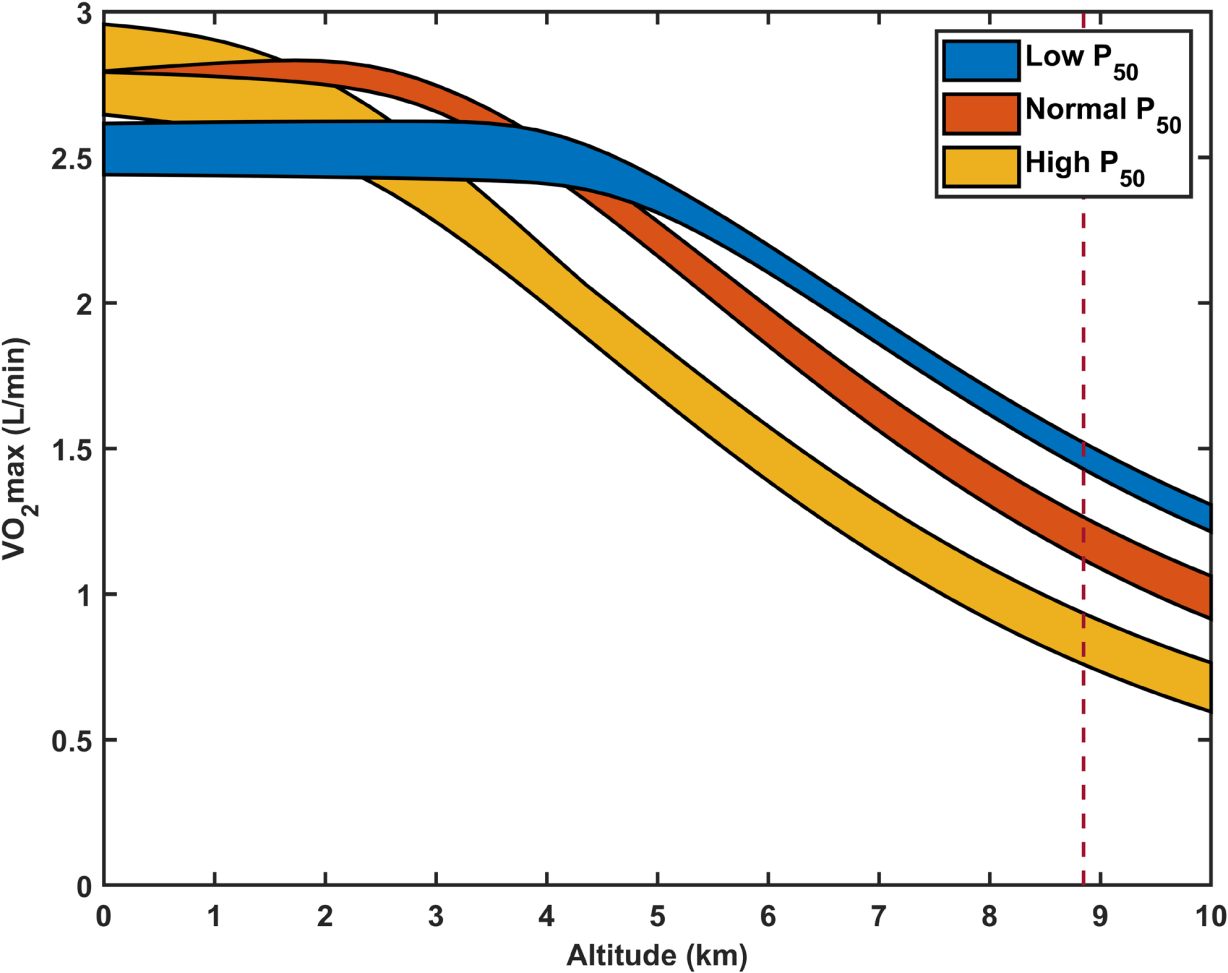
The dependence of predicted maximum oxygen uptake and utilization (*V̇*O2max) on hemoglobin‐oxygen affinity (P_50_) and altitude. Data are depicted for several cases of hemoglobin‐oxygen affinity (low P_50_ in blue, normal P_50_ in red, and high P_50_ in yellow) with variable hemoglobin concentrations to account for potential differences in the hematological response to extreme altitude sojourn indicated in Table 1. These parameters are derived for a given tissue oxygen demand that corresponds with sea‐level *V̇*O_2_max. The elevation associated with the summit of Everest is depicted by the dashed vertical line. Parameters corresponding to altitudes above 8400 m are derived from the extrapolation of oxygen transport parameters in Grocott et al. (2009). *P*_50_, oxygen tension at which 50% of hemoglobin is saturated with oxygen.

The predicted *V̇*O_2_max as a function of altitude is depicted in Figure 5 for the cases of low, normal, and high *P*_50_. As expected, *V̇*O_2_max decreases with increasing altitude in all cases. However, the variation of *V̇*O_2_max with altitude is markedly dependent on hemoglobin‐oxygen affinity. In the case of high *P*_50_, the predicted *V̇*O_2_max at sea level is greater than predicted values for normal *P*_50_, but markedly lower at altitudes above ~2500 m. Conversely, in the case of low *P*_50_, *V̇*O_2_max is lower than values predicted for normal *P*_50_ at sea level, but greater at altitudes above ~4500 m.

**FIGURE 6:**
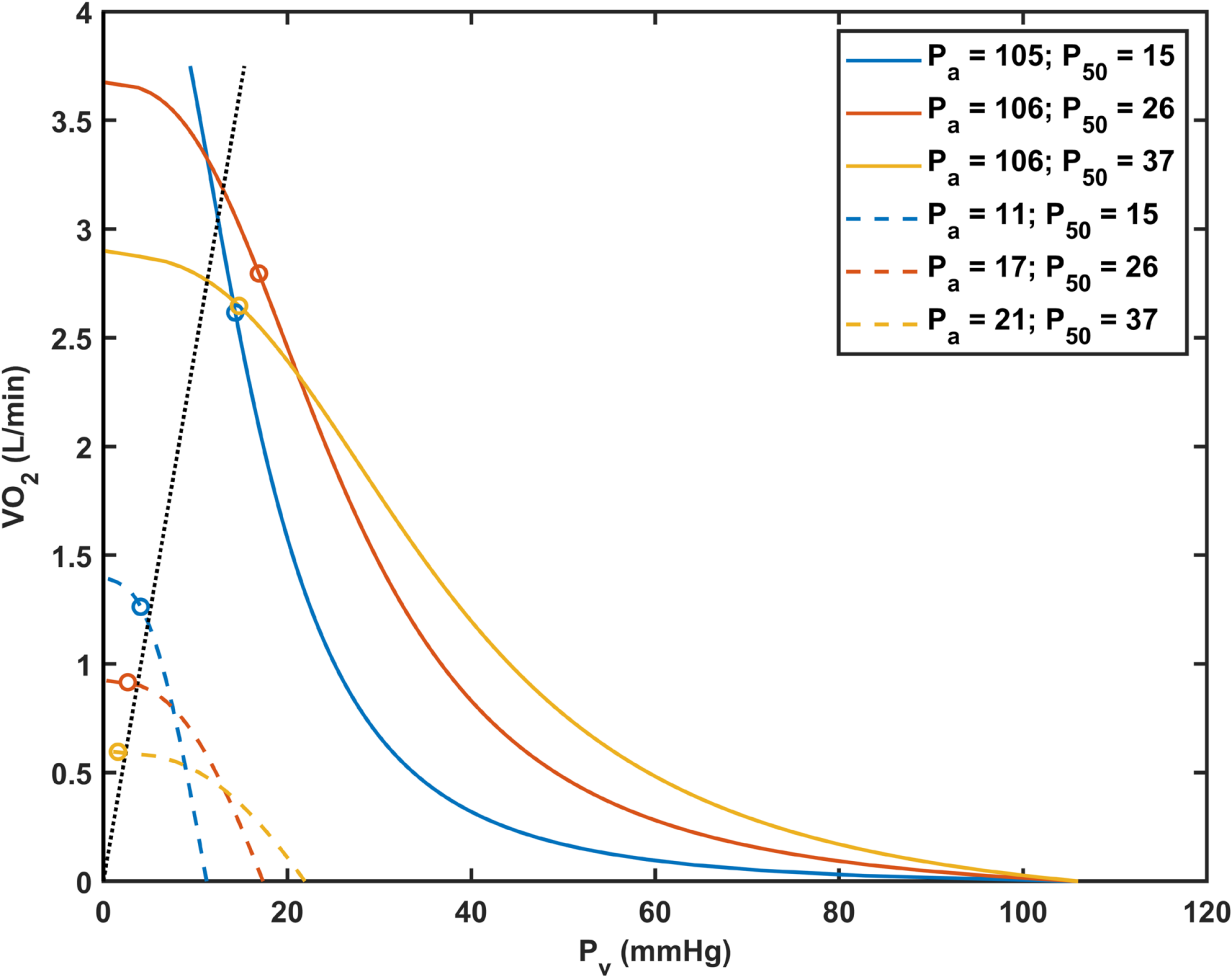
Predicted oxygen uptake and utilization (*V̇*O2) presented as a Wagner diagram for cases of low, normal, and high P50. Data for each group are presented at sea‐level (solid lines) and an altitude of ~8850 m (summit of Mt. Everest, represented by dashed lines). The *V̇*O_2_max value is depicted by the intersection between convective oxygen transport (curved lines obtained via Fick principle) and diffusive oxygen transport (dotted line passing through the origin). Open circles plotted on curved lines denote the predicted *V̇*O_2_max as determined using Michaelis–Menten kinetics. *P*_a,_ arterial oxygen tension; *V̇*O_2_, oxygen uptake and utilization, *P*_50_; oxygen tension at which 50% of hemoglobin is saturated with oxygen. Values of *P*_a_ and *P*_50_ are in units of mmHg.

Predictions of *V̇*O_2_max from the present model are presented in Figure 6 together with lines and curves representing limitations on oxygen utilization according to the Wagner diagram. At sea level, *V̇*O_2_max shows small variations with *P*_50_, with a slight advantage at normal *P*_50_. At an altitude of ~8850 m, convective oxygen delivery is greatly reduced and shows a strong inverse dependence on *P*_50_. The higher rates of convective oxygen delivery at low *P*_50_ result from two factors: higher arterial oxygen saturation (~45%, vs. ~30% for high *P*_50_) and higher hemoglobin values (~50% greater than for high *P*_50_).

## 4 DISCUSSION

### 4.1 Physiological implications

*V̇*O_2_max is determined by convective and diffusive oxygen transport, both of which are influenced by alterations in hemoglobin‐oxygen affinity (Hebbel et al., 1977; Webb, Elshaer, et al., 2022b). Specifically, high hemoglobin‐oxygen affinity tends to enhance pulmonary oxygen uptake, particularly when alveolar oxygen tension is low, increasing convective oxygen transport. On the contrary, high hemoglobin‐oxygen affinity implies a lower blood oxygen tension for a given level of oxygen saturation, such that the driving force for oxygen diffusion from blood to tissue is reduced. The relative influences of these two competing effects of high hemoglobin‐oxygen affinity (low *P*_50_) on *V̇*O_2_max cannot easily be discerned by qualitative arguments. Therefore, we investigated this relationship using a mathematical model of oxygen transport that includes both pulmonary and systemic circulation and considers the effects of both convective and diffusive oxygen transport. The model also considers the influence of high altitude on oxygen availability and uptake in the lungs.

Previous analyses of effects of *P*_50_ on oxygen transport at extreme altitude suggested that *V̇*O_2_max is insensitive to *P*_50_ over a considerable range (Bencowitz et al., 1982; Wagner, 1997). The present model differs from those analyses in two significant respects. First, it includes effects of variations in hemoglobin levels in individuals with altered *P*_50_, which results in increased convective oxygen delivery in the case of low *P*_50_. Second, it takes into account the nonlinear Michaelis–Menten kinetics of oxygen utilization as a function of tissue *P*O_2_, representing the finite rate of mitochondrial oxygen consumption when oxygen is not rate‐limiting. Both of these effects result in increased predictions of *V̇*O_2_max at extreme altitude for reduced *P*_50_, and account for the apparent discrepancy with the earlier work.

The diffusive limitation of oxygen transport, Equation 11, is computed assuming a uniform rate of oxygen consumption throughout the tissue. In contrast, the FickMM model allows for variations of oxygen levels and oxygen consumption rates in the tissue, including possible hypoxic regions. The resulting estimates of *V̇*O_2_max at altitude are slightly higher than those obtained from the intersections of the diffusive limitation line and the convective delivery curves, as shown in Figure 6. This difference is most evident in the case of low *P*_50_. The diffusive limitation line shown in Figure 6 is based on the assumption that *P*O_2_ values approach zero only at the point in the tissue furthest from the distal end of the supplying capillary. If oxygen demand is further increased, overall oxygen consumption can increase beyond the value implied by diffusive limitation, even if some regions of tissue are hypoxic (McGuire & Secomb, 2001).

Humans at high altitude experience a range of acute (dehydration, alkalosis, hypocapnia) and chronic (training, acclimatization) effects, both of which may be associated with variations in *P*_50_ and hemoglobin concentration (Mairbaurl & Weber, 2012; Monge & Leon‐Velarde, 1991; Windsor & Rodway, 2007). Because these effects are not widely characterized among humans with hemoglobin mutations, we considered a range of cases for each case of hemoglobin‐oxygen affinity (low, normal, and high *P*_50_). Thus, the predicted *V̇*O_2_max is provided with a range of values for each altitude and case examined, providing an indication of the sensitivity of the model to these potential variations in *P*_50_ and hemoglobin concentration. Individual variations in physiological parameters such as capillary density and lung diffusing capacity, as well as potential alterations of these parameters during extreme altitude sojourn, may be substantial and would obviously affect the predictions of this model. However, the primary trends in *V̇*O_2_max as a function of altitude are likely to remain similar even if baseline values are notably different. Other parameters of oxygen transport, such as lung diffusing capacity and the respiratory quotient, may vary with altitude but are assumed to be constant in the model. Additionally, the effects of non‐muscle blood flow on overall oxygen transport are not considered, as the entire cardiac output is assumed to be directed to the skeletal muscle during maximal exercise.

Our results revealed that at low altitudes, where atmospheric pressure is more than sufficient to cause nearly complete saturation of hemoglobin, a low *P*_50_ does not confer an advantage in terms of oxygen utilization since convective transport is sufficient to supply skeletal muscle. At high altitudes, however, a low *P*_50_ increases convective oxygen delivery due to higher oxygen saturation values, despite the diffusion limitation resulting from lower blood oxygen tension. This improved oxygen delivery allows for better preservation of *V̇*O_2_max at high altitudes. In summary, a low *P*_50_ leads to a reduced driving force for oxygen diffusion from blood to tissue at low altitudes yet increased convective oxygen delivery at high altitudes. These two competing tendencies approximately cancel at an altitude of ~4500 m such that high hemoglobin‐oxygen affinity confers an advantage at higher altitudes.

In the results presented here, the effects of capillary density are not explicitly considered under the approximation that venous oxygen tension is representative of tissue oxygen tension. More detailed calculations show that high capillary densities can lead to greater tissue oxygen tensions values than assumed here. A high capillary density may facilitate the advantage conferred by a low *P*_50_ due to decreased diffusion limitation. Conversely, a low capillary density may negate the advantage of a low *P*_50_ at high and extreme altitude because oxygen delivery would then be limited by reduced muscle diffusing capacity.

### 4.2 Practical applications

Studies in comparative physiology show a wide range of adaptations to altitude, some of which have supported that an increase in hemoglobin‐oxygen affinity is likely beneficial for species adapted to high and extreme altitude (Natarajan et al., 2018; Storz, 2007; Storz et al., 2010). Across species, multiple factors including evolutionary pressures may influence the observed adaptations in hemoglobin‐oxygen affinity. Further detailed investigation of this topic in terms of convective versus diffusive oxygen transport limitations would be appropriate, and the theoretical approach developed here may be applicable to such studies.

Pharmacological agents have been developed that can alter *P*_50_ in healthy individuals (Henry et al., 2021; Safo & Kato, 2014; Woyke et al., 2021). Although these agents are mainly investigated for treatment of sickle‐cell disease, they have also been used in healthy individuals (Stewart et al., 2021, 2020). According to the present results, decreasing the *P*_50_ has significant effects on blood oxygenation and *V̇*O_2_max at altitude, some of which may prove beneficial depending on the environmental context. For instance, pharmacologically decreasing the *P*_50_ may have an ergogenic effect at high and extreme altitudes by increasing arterial blood saturation and improving convective oxygen delivery. This raises the possibility that such agents could be used for “blood doping” in competitive sports. In military operations at high and extreme altitudes, environmental conditions may limit physical performance and cognitive function (McLaughlin et al., 2017). Pharmacological reduction in *P*_50_ may increase hypoxia tolerance (Dufu et al., 2021) and prevent decrements in physical performance (Stewart et al., 2021). However, further work is needed to examine the advantages or disadvantages of pharmacologically altering *P*_50_ in healthy individuals in various contexts. The present model may be useful for predicting the change in *P*_50_ at a given altitude that maximizes the ergogenic effect.

### 4.3 Limitations

A major simplification of this model is the use of venous *P*O_2_ as a measure of tissue *P*O_2_ for the purpose of calculating oxygen consumption according to Michaelis–Menten kinetics. The rationale for this assumption is that venous *P*O_2_ typically lies within the range of the minimum and maximum tissue *P*O_2_. As shown in Figure 3, oxygen consumption rates calculated under this assumption show reasonable agreement with more detailed calculations using a Krogh cylinder model. This approach avoids the need to specify the geometry of the capillary network, since such detailed information is generally not available. However, the limitation of this approach is that it does not include the effects of capillary network geometry.

Previous studies have indicated that the Bohr effect (pH dependent change in the *P*_50_) may play a notable role in the determination of *V̇*O_2_max (Severinghaus, 1994). However, this effect was not considered in the present model. Because the magnitude of the Bohr effect at extreme altitudes is not known, it was excluded to facilitate comparisons across altitudes. Given that the Bohr effect is generally preserved among humans with hemoglobin mutations (Boyer et al., 1972), its effect on *V̇*O_2_max values would likely be unidirectional and comparable between the groups examined. Additionally, past investigations have described aberrations in metabolic processes during exercise among humans with low *P*_50_ and suggested that skeletal muscle and mitochondrial adaptations may compensate for the blunted oxygen offloading (Wranne et al., 1983). At extreme altitude, however, *V̇*O_2_max is severely limited by the reduced oxygen availability, such that changes in maximal mitochondrial oxygen consumption are unlikely to affect *V̇*O_2_max. Therefore, the present model assumes similar mitochondrial function between groups with normal and altered *P*_50_.

## 5 CONCLUSION

The presented analyses leverage experimental data among humans with hemoglobin mutations to predict blood oxygenation and *V̇*O_2_max as a function of hemoglobin‐oxygen affinity and altitude. We posit that high hemoglobin‐oxygen affinity leads to improved blood oxygenation and better preserved *V̇*O_2_max values at extreme altitudes compared to values associated with normal hemoglobin‐oxygen affinity. Additionally, we provide theoretical estimates for *V̇*O_2_max as a function of altitude among humans with mutations causing low hemoglobin‐oxygen affinity, which has yet to be examined experimentally.

## AUTHOR CONTRIBUTIONS

Kevin L. Webb and Tuhin K. Roy conceived the presented idea. All author contributed to the methodological design of this work. Kevin L. Webb, Tuhin K. Roy, and Timothy W. Secomb contributed to model development, analyses, and data visualization. Kevin L. Webb and Tuhin K. Roy constructed the initial manuscript draft. All authors contributed to manuscript revising and have approved the final submission.

## FUNDING INFORMATION

This work was funded by the National Institutes of Health grant R35‐HL139854 (M.J.J.).

## CONFLICT OF INTEREST STATEMENT

The authors have no conflict of interest to declare.

## ETHICS STATEMENT

The presented study was exempt from obtaining IRB approval and does not present novel data pertaining to human nor animal subjects.

## Data Availability

All pertinent data are presented within the manuscript. All code used to perform calculations will be shared upon reasonable request.
